# Poisoning with Secale Cornutum

**Published:** 1851-01

**Authors:** 


*Poisoning with Secale Cornutum*—Dr. PRATSCHKE was called on the 12th October, 1844, to a woman who, with her three children, had been taken ill the day before. Five days previously they had eaten bread which contained a large proportion of blighted corn. The mother, forty years of age, complained of uneasiness and heaviness in the head, oppression at the stomach, loss of appetite, and diarrhœa; but she did not feel ill enough to take to her bed. The eldest daughter, eighteen years of age, complained of a violent sense of burning in the hands and feet, and especially in the fingers and toes, which were bent and stiff. The lips were retracted so as to expose the teeth; the tongue white and moist; the skin dry and cool; the pulse 90, and small: the patient was very restless, and expressed urgent thirst; the abdomen was soft; the bowels acting; the urine pale. She died on the following day in violent convulsions.

The second daughter, seven years old, had the same affection of the lower extremities, which in her case occurred periodically: her appetite was good: she had also diarrhœa.

The third child, four years old, suffered only from diarrhœa.

An emetic was given to all in the first place; camphor was afterwards administered.

The mother suffered for a few days from tetanic cramps, and continued to complain of great anxiety, loss of appetite, and diarrhœa. After the exhibition of valerian, muriate of ammonia, and ipecacuanha, followed by extract of nux vomica, these symptoms disappeared, but were succeeded by anesthesia of the soles of the feet.

The second daughter was restored to health, with the exception that she had not recovered the use of her legs.

The youngest child suffered from tetanic cramps for several days; and considerable stiffness of the limbs remained, so that she frequently fell in walking. This subsided under the use of aromatic baths.—*Lond. Med. Gaz., from Casper's Wochenschrift.*

